# Alpha7 Nicotinic Acetylcholine Receptor Mediates Nicotine-induced Apoptosis and Cell Cycle Arrest of Hepatocellular Carcinoma HepG2 Cells

**DOI:** 10.15171/apb.2020.008

**Published:** 2019-12-11

**Authors:** Khalil Hajiasgharzadeh, Mohammad Hossein Somi, Behzad Mansoori, Mohammad Amin Doustvandi, Fatemeh Vahidian, Mohsen Alizadeh, Ahad Mokhtarzadeh, Dariush Shanehbandi, Behzad Baradaran

**Affiliations:** ^1^Immunology Research Center, Tabriz University of Medical Sciences, Tabriz, Iran.; ^2^Liver and Gastrointestinal Diseases Research Center, Tabriz University of Medical Sciences, Tabriz, Iran.; ^3^Department of Immunology, Faculty of Medicine, Tabriz University of Medical Sciences, Tabriz, Iran.

**Keywords:** Alpha7 nicotinic acetylcholine receptor, Small interfering RNA, Nicotine, HepG2, Apoptosis

## Abstract

***Purpose:*** The cytotoxic properties upon treatment with nicotine have been reported in several studies, but the underlying mechanisms remain not fully defined. The alpha7 nicotinic acetylcholine receptor (α7nAChR) is one of the important nicotinic receptors, which nicotine partly by binding to this receptor exerts its effects. The current study aimed to investigates the influences of nicotine on cellular proliferative and apoptotic activities and tried to determine the involvement of α7nAChR in these functions.

***Methods:*** Human hepatocellular carcinoma (HepG2) cell line was used to determine the individual or combined effects of treatments with nicotine (10 μM) and specific siRNA (100 nM) targeting α7nAChR expression. The MTT assay, DAPI staining assay, and flow cytometry assay were applied to measure the cell viability, apoptosis and cell cycle progression of the cells, respectively. In addition, the changes in the mRNA level of the genes were assessed by qRT-PCR.

***Results:*** Compared to control groups, the cells treated with nicotine exhibited significant dosedependent decreases in cell viability (log IC50 = -5.12±0.15). Furthermore, nicotine induced apoptosis and cell cycle arrest especially at G2/M Phase. The qRT-PCR revealed that nicotine increased the mRNA levels of α7nAChR as well as caspase-3 and suppressed the expression of cyclin B1. Treatment with α7-siRNA abolished these effects of nicotine.

***Conclusion:*** These experiments determined that upregulation of α7nAChR by nicotine inhibits HepG2 cells proliferation and induces their apoptosis. These effects blocked by treatment with α7-siRNA, which indicates the involvement of α7nAChR pathways in these processes.

## Introduction

The smoking habit is a health disparity and a well-known risk factor for different diseases including cancers, but further researches are needed to be conducted on elucidating the mechanisms underlying the effects of cigarette use.^1^ Nicotine as a well-documented component of cigarette smoking is an addictive substance of tobacco and poses several health hazards.^2^ This compound exerts its consequences by binding to nicotinic acetylcholine receptors (nAChRs) that are ligand-activated ion channels.^3^ Among different subtypes of nAChRs, alpha7-subtype of nAChR (α7nAChR) appears to be of special significance in cellular functions. This receptor functionally expressed by a variety of human normal and cancer cell and tissues such as liver cancer.^4^ It modulates numerous cancer-related properties in most of the cancers and additionally plays a crucial role in the regulation of inflammation through the cholinergic anti-inflammatory pathway in various pathological conditions.^5,6^ Such diverse impacts that initiate from this receptor may have significant influences in determining the consequences of different cancers.

The list of malignancies that are related to nicotine exposure is increasing and involves many different types of cancers.^7^ Among them, hepatocellular carcinoma (HCC), as a common type of liver cancer, is one of the most dangerous and difficult-to-cure malignancies in the world.^8^ Several epidemiologic studies have reported that tobacco smoking is causally associated with HCC development.^9^ The consumed nicotine is broadly metabolized in the liver to produce various types of metabolites.^10^ In a recent study, it has been shown that in humans, the greatest amount of α7nAChR recognized in the liver and this finding demonstrated the importance of α7 receptor-related functions in this organ.^11^ Nicotine as a double-edged sword exerts diverse physiological and pathological effects in the liver and it may modify the HCC initiation and progression. However, our knowledge about the role of nicotine and α7nAChR in the development of HCC is very limited and the role and mechanisms by which nicotine influences this cancer remain unclear. There will be opportunities for the identification of novel treatments and prevention options in the future, if follow-up research on the impacts of nicotine through α7nAChRs continues. In this study, we determined the effects of treatment with nicotine on human hepatocellular carcinoma (HepG2) cell line and aimed to investigate the involvement of α7nAChR in its effects.

## Materials and Methods

### Main material and reagents

HepG2 cell line was obtained from National Cell Bank of Iran (Pasteur Institute of Iran, Tehran, Iran). Cell culture substances, Roswell Park Memorial Institute (RPMI) 1640 medium, fetal bovine serum (FBS), trypsin/EDTA and penicillin/streptomycin mixtures were purchased from Gibco Co. (Gibco, Carlsbad, CA, USA). The rest of the materials were bought from Santa Cruz Biotechnology (Santa Cruz, CA, USA), unless otherwise specified in the text. The cells were sustained in RPMI medium with 10% FBS and routinely cultured at 37 °C with 5% CO_2_ and were used in the logarithmic phase of growth in all tests according to our previous study.^12^ Each experiment was repeated three times.

### MTT assay

HepG2 cells were cultured at a density of 15×10^3^ cells per well in 96-well culture plates. Nicotine (SC-203161) in various concentrations (10-^8^ M to 10-^2^ M) was added to the culture and incubated for 72 h. To determine the concentration-dependent cytotoxicity of nicotine, the MTT (3-(4,5-Dimethylthiazol-2-yl)-2,5-diphenyltetrazolium Bromide) assay was used as previously described.^13^ Briefly, the MTT at a concentration of 2 mg/mL was added to the wells and after that incubated for 4 h at 37°C. After removal of the media, 200 μL dimethyl sulfoxide (DMSO) was added to the wells. The values of optical density (OD) of the cells were evaluated at 570 nm with an ELISA Reader (Sunrise RC, Tecan, Switzerland). The results were shown as percentages of the control groups.

### siRNA transfection

The siRNA targeting human α7nAChR (sense: 5′‐ CCAGACAUUCUCCUCUAUA‐3) and the negative control siRNA were purchased from Microcynth (AG, Switzerland). Cells were transfected with these siRNAs following the manufacturer’s guidelines. In brief, 2×10^5^ cells were seeded at 6-well plates in RPMI-10% FBS medium. The siRNAs at a final concentration of 100 nM in all experiments were transfected into the cells by using nanoparticles according to our previous studies.^14^ The siRNAs and nanoparticles were diluted in dilution buffer and incubated for 20 min at room temperature. Afterward, the siRNAs were added to the wells with Opti-MEM solution. The plates were then incubated for additional 6 h at 37˚C in a CO_2_ incubator. Following that, RPMI-20% FBS medium was added to the wells containing transfected cells. After 48 h of incubation, the suppression of α7nAChR gene expression was determined by quantitative real-time PCR (qRT-PCR) method.

### RNA isolation, cDNA synthesis, and qRT-PCR

The gene expression of α7nAChR and other apoptosis and cell cycle-related genes were analyzed by qRT-PCR. Briefly, 2×10^5^ cells were seeded into 6-well plates one day before the start of the nicotine and siRNA treatments. Total RNA was isolated from the cells using TRIzol (Riboex, Gene All Biotechnology, Seoul, Korea). The total RNA purity and integrity was confirmed by using a NanoDrop (Thermo Scientific, USA). Then 1μg total RNA was reverse transcribed into cDNA (Biofact, South Korea). The qRT-PCR was performed using light cycler 96 (Roche Diagnostics, Mannheim, Germany) and reported by the 2^−ΔΔCT^ method. Glyceraldehyde 3‐phosphate dehydrogenase (GAPDH) was used as an internal control. The primers sequences for α7nAChR and other genes were obtained from Sinaclon (Tehran, Iran) and listed in Table 1.

**Table 1:** The sequence of primers for alpha7 nicotinic acetylcholine receptor (α7nAChR), caspase-3, cyclin B1, and GAPDH genes

**Genes**		**Sequences**
α7nAChR	Forward	5´ CGCCACATTCCACACTAACG 3´
	Reverse	5´ AGACCAGGACCCAAACTTCAG 3´
Caspase-3	Forward	5´ TGTCATCTCGCTCTGGTACG 3´
	Reverse	5´ AAATGACCCCTTCATCACCA 3´
Cyclin B1	Forward	5´ GGTTGG GTCGGCCTCTACCT 3´
	Reverse	5´ AGCCAGGTGCTGCATAACTGGAA 3´
GAPDH	Forward	5´ CAAGATCATCAGCAATGCCTCC 3´
	Reverse	5´ GCCATCACGCCACAGTTTCC 3´

### Apoptosis assays

#### Annexin/PI assay

The apoptosis of HepG2 cells was assessed by flow cytometry assay using propidium iodide (PI) fluorescence staining. To estimate the percentage of apoptosis of the cells, they were seeded in the 6-well plates at a density of 2×10^5^ cells per well. After 72 h of the treatment with nicotine and 48 h of the siRNA transfection, the cells were stained with an Annexin V‐FITC/PI staining assay kit (EXBIO, Vestec, Czech Republic). By using flow cytometry instrument (MACS Quant 10; Miltenyi Biotech, GmbH, Germany) the rate of apoptotic cells was measured and obtained data were analyzed using the package of FlowJo software (Treestar, Inc., San Carlos, CA).

#### DAPI staining assay

To evaluate the effect of chronic nicotine exposure and gene silencing of α7nAChR on chromatin fragmentation, DAPI (4′,6‐diamidino‐2‐phenylindole) staining was performed. For this aim, approximately 15×10^3^ of the cells were seeded into 96-well plates. After that, the cells were silenced alone or in a combination with pretreatment with nicotine for 24 h. After the fixation of the cells with 5% paraformaldehyde for 4 h, In the next step, the cells were incubated with Triton X-100 (0.1%) for 5 min and then were stained with DAPI (0.1%) for an additional 10 min. Ultimately, the cells were observed by using an imaging fluorescence microscope system (Cytation 5, Biotek, USA).

#### Cell cycle analysis

For determining the cell cycle arrest properties, HepG2 cells were seeded at 6-well plate and then treated with nicotine for 24 h and α7-siRNA for 48 h, respectively. The cells were collected by centrifugation and were incubated with PI using flow cytometric kits (EXBIO, Vestec, Czech Republic) according to the manufacturer’s recommendations.^15^ In brief, the cell plates were dissolved in a mixture of PBS and RNase A solution and incubated for 30 min. Then 1 mL of the Tris buffer solution was blended with 100 mL of PI solution and added to each well. Ultimately, after 10 min incubation time the cell cycle analysis was carried out by a flow cytometry system (MACS Quant 10; Miltenyi Biotech, GmbH, Germany).

#### Statistical analyses

All data are shown as the mean ± SEM. Statistical significance of differences between variables with normal distribution was assessed via one-way ANOVA followed by Tukey post hoc test by using GraphPad Prism 6 software (San Diego, CA, USA). Two-way ANOVA was used when the effect of the two variables was assessed. The *P* values smaller than 0.05 were considered statistically significant.

## Results

### Analysis of cell viability

**Figure 1:**
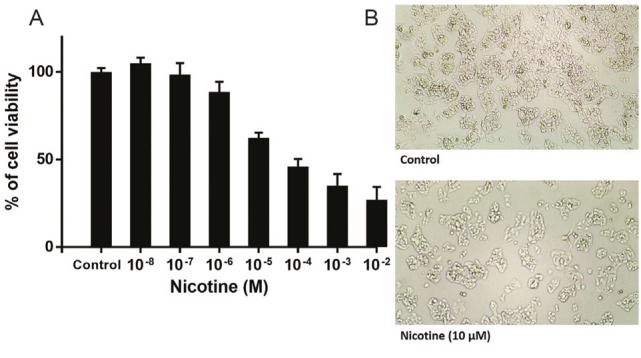
(A) Nicotine leads to the inhibition of the proliferation of HepG2 cells in dose-dependent patterns (log IC50 = -5.12±0.15). Data are shown as Mean ± SEM and all of the experiments were repeated three times. (B) In IC50 concentration of nicotine, the cells underwent a significant decrease in cellular density and increased toxicity was observed at a higher concentration of nicotine.

The MTT results indicate that 72 h nicotine treatment significantly reduced the viability of HepG2 cells in a concentration-dependent fashion (log IC50 = -5.12±0.15) (Figure 1A). Increased toxicity was observed at a higher concentration of nicotine. Based on this result, we selected IC50 doses of nicotine (10 µM) for subsequent experiments. In this concentration of nicotine, the cells underwent a significant decrease in cellular density (Figure 1B).

### Nicotine upregulates α7nAChR expression, an effect that blocked by α7-siRNA transfection

**Figure 2:**
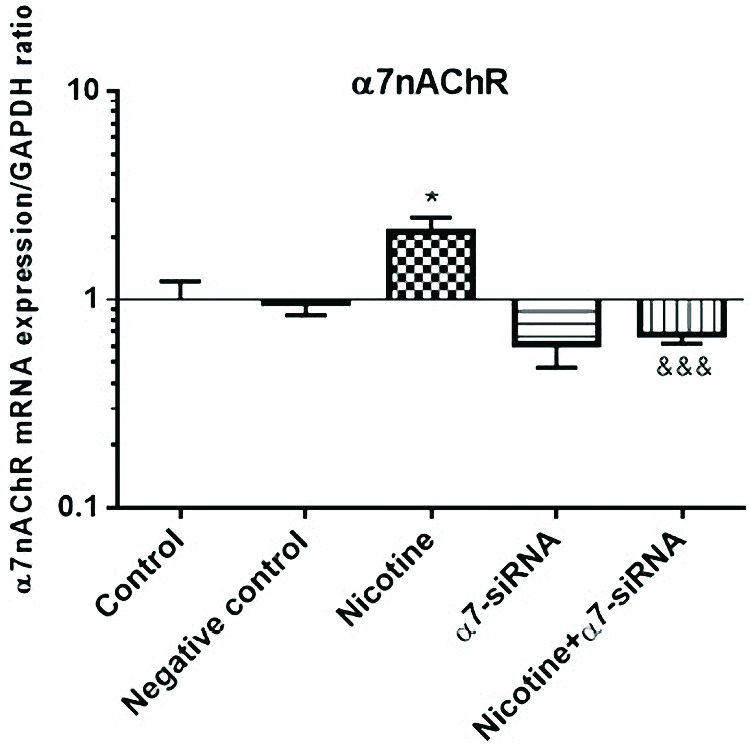
The mRNA level of α7nAChR was upregulated after treatment with nicotine (10 μM) as compared with non-treated cells. This effect was blocked by α7-siRNA transfection. To determine the differences between the statistical significance of the groups, Student’s T and ANOVA tests were performed. Negative control siRNA has no significant effect on α7nAChR mRNA expression. The results were normalized with the GAPDH housekeeping gene mRNA level. *P< 0.05 in comparison with the control group. ^&&&^*P*<0.001 in comparison with nicotine-treated group.

The HepG2 cells were examined for α7nAChR gene expression using RT-PCR. We achieved that after 72 h treatment with nicotine (10 μM) the mRNA level of α7nAChR was upregulated as compared with non-treated cells (Figure 2). The efficiency of siRNA in down-regulation of α7nAChR expression was examined in combined nicotine and α7-siRNA treatment groups. The results indicated that the increased effect of nicotine on α7nAChR expression was blocked by treatment with α7-siRNA transfection (Figure 2). Negative control siRNA has no significant effect on α7nAChR mRNA expression. The results were normalized with the GAPDH housekeeping gene mRNA level.

### Effect of α7-siRNA on apoptosis of HepG2 cells

**Figure 3:**
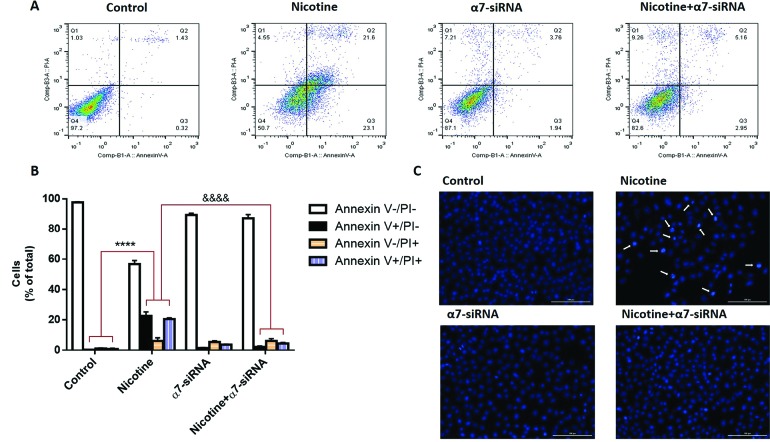
(A) By utilizing the fluorescence‐activated cell sorting analysis and annexin V/PI assay, the portion of apoptotic cells were separated in HepG2 cells that incubated with nicotine, α7-siRNA or combination of them. (B) The analysis indicated that nicotine strongly promotes apoptosis of HepG2 cells. ****P <0.0001 in comparison with control group. The siRNA-mediated knockdown of α7nAChR in these cells inhibits the proapoptotic effects of nicotine. &&&&P <0.0001 in comparison with nicotine group. (C) DAPI staining also validated the fragmentation of chromatin which occurs in the processes of apoptosis and increased in nicotine-treated cells compared with non-treated cells. This effect blocked in α7-siRNA transfected cells.

The effects of nicotine and α7-siRNA on apoptosis of HepG2 cells were determined by flow cytometry (Annexin V and PI staining) assay. By using this technique and fluorescence‐activated cell sorting analysis, the portion of apoptotic cells was analyzed and quantified in the cells that incubated with nicotine or α7-siRNA or combination of them (Figure 3A). In this technique, viable cells are both annexin and PI negative (annexin V-/PI-), while early apoptotic cells are annexin V+/PI-. Also, the cells in late apoptosis are both Annexin V and PI positive and necrotic cells stain with PI only (V-/PI+).^16^ The results indicated that nicotine strongly promoted both early and late apoptosis of the cells but α7-siRNA inhibited these changes (Figure 3B). In addition to this, DAPI staining verified that the chromatin fragmentation in the processes of apoptosis is enhanced in nicotine-treated cells compared with non-treated control cells. Similar to flow cytometry results, these effects blocked in α7-siRNA transfected cells (Figure 3C).

### Effect of α7-siRNA on cell cycle progression of HepG2 cells

**Figure 4:**
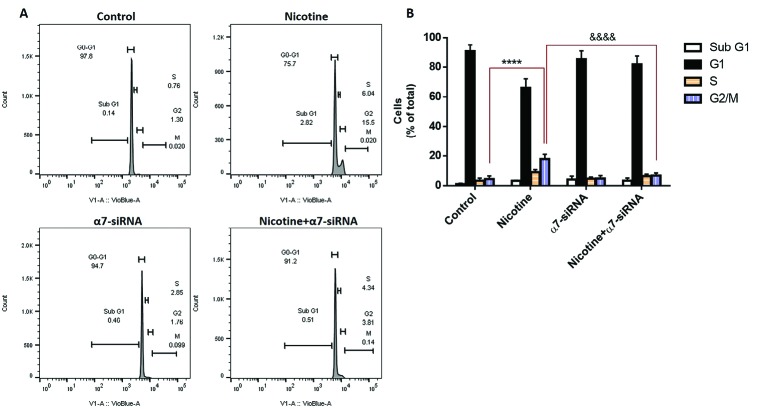
(A) The cell cycle distribution was determined after treatment with nicotine, α7-siRNA or combination of them. (B) Flow cytometry analysis showed that nicotine inhibited cell cycle progression by inducing G2/M phase arrest. These increases were reduced when the cells incubated with α7-siRNA. ****P< 0.0001 in comparison with control group. ^&&&&^*P*<0.0001 in comparison with nicotine group.

We investigated whether nicotine influences the arrest of the cell cycle in HepG2 cells. For this purpose, flow cytometry analysis was performed to differentiate the diverse phases of the cell cycle (Figure 4A). The results showed that nicotine inhibited cell cycle progression by especially inducing G2/M phase arrest. This increased response in cell cycle arrest was blocked when the cells were incubated with α7-siRNA, which indicated the pivotal role of the expression of this receptor in nicotine-induced cell cycle arrest of HepG2 cells (Figure 4B).

### Expression analysis of caspase-3 and cyclin B1 mRNA levels

**Figure 5:**
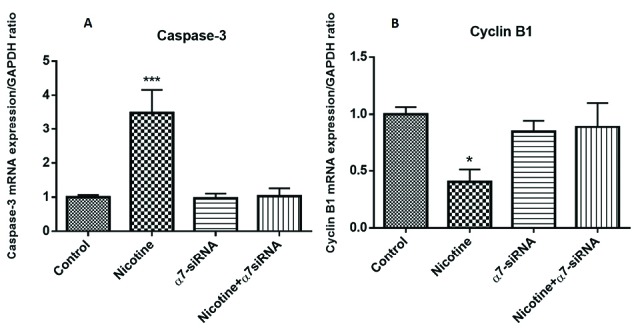
(A) Nicotine caused the greatest increase in the gene expression of caspase-3 in the HepG2 cells. ***P< 0.001 in comparison with the control group. (B) Incubation of nicotine induces down-regulation of cyclin B1 expression. *P< 0.05 in comparison with the control group. These observed effects of nicotine on caspase-3 and cyclin B1 mRNA levels were abolished by transfection with α7-siRNA.

Assessment of apoptosis-related gene revealed that nicotine caused the greatest increase in the expression of caspase-3 in the cells (Figure 5A). In addition to this, cyclin B1 expression as a cell cycle progression-related gene was examined. This gene is a regulatory gene and playing the most pivotal role in the cell cycle progression and transition of the G2/M phase.^17^ The results indicated that incubation of nicotine induced down-regulation of cyclin B1 expression (Figure 5B). These observed effects of nicotine on caspase-3 and cyclin B1 mRNA levels were abolished by transfection with α7-siRNA, which provided other evidence about the involvement of α7nAChR in the control of various pathways inducing the mitochondrial type of apoptosis.

## Discussion

Nicotine, which is an important component of cigarette smoking, may be responsible for various cancers initiation, progression, and therapy responses.^18^ This compound has been shown to influence an extensive variety of biological functions ranging from gene expression, oxidative stress, DNA damage, apoptosis, proliferation, angiogenesis, and regulation of hormone secretion.^18,19^ There is controversy in the literature about nicotine-induced toxicity in exposed subjects.^20^ It is well established that nicotine treatment results in oxidative stress that is associated with multiple alterations of cell structure and function.^21^ Also, accumulating evidence indicates that exposure to nicotine can result in the activation of intracellular signaling pathways that are known to induce apoptosis.^22-24^ In this context, to defend against the harmful effects of this substance, the protective effects of many natural and pharmacologic agents on nicotine-induced toxicity have been investigated in numerous studies.^25,26^ Kim and colleagues revealed that α7nAChR contributes to the proapoptotic effects of nicotine in periodontal cells.^27^ In this study, the proapoptotic effects of nicotine were abolished by the pretreatment of α-bungarotoxin, a selective antagonist of α7nAChR, which highlighted the key role of α7nAChR in the modulation of nicotine-induced apoptosis.^27^ Conversely to these findings, in some other studies nicotine induced the antiapoptotic and proliferative effects in different cell types.^28-30^ Therefore, the effect of nicotine on apoptosis may be complicated in various parts of the body. For instance, Jalili and colleagues have shown that in the heart and lung, nicotine caused a significant decrease in caspase-3 mRNA level compared to the control group.^31^ However, in the kidney and liver, the results were significant increases in caspase-3 mRNA level which indicates the different responses of organs to the nicotine exposure.^31^ It is well recognized that apoptosis plays important roles in an extensive variety of physiologic processes and is a major feature of the biology of malignant diseases. It should be mention that, in the liver tissue, enhanced hepatocyte apoptosis and death could result in induced compensatory proliferation.^32^ In most cases, this event eventually leads to the appearance of HCC.^33^ Furthermore, it is known that the growth and proliferation of the progenitor or stem cells are stimulated via signals that are released from apoptotic cells.^34^ Li and colloquies have shown in an animal model that wound healing and liver regeneration after partial hepatectomy are dependent on Caspase-3 because targeted inactivation of this caspase inhibits these regenerative processes.^35^ By considering these facts, we assumed that nicotine by dysregulation of the apoptotic processes may involve in the etiology of liver cancer.

**Figure 6:**
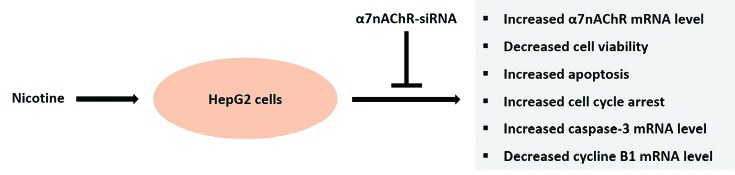
Treatment of the HepG2 cells with the α7nAChR specific siRNA blocks nicotine-induced proliferative and apoptotic effects.

The epidemiological positive correlation between cigarette smoking and liver cancer has been demonstrated in previous studies.^9^ Among different subtypes of nicotinic receptors, α7nAChR is one of the highly expressed receptors in the liver.^4^ In the present study, the effect of nicotine on the viability of cultures of HepG2 cells was analyzed and we provide functional evidence that nicotine induced proapoptotic gene caspase-3 expression and increased apoptosis through the α7nAChR in HepG2 cells (Figure 6). It has been shown that nicotinic receptors activation can induce hepatocyte proliferation, but only in the presence of hepatic nonparenchymal cells.^36^ Therefore, the role of other liver resident cells such as Kupffer cells and hepatic stellate cells in the diverse functions of α7nAChR in this organ should be carefully considered when investigating the toxic or proliferative effects of nicotine in the liver. In addition to apoptosis, it is clear that cell cycle arrest mediates apoptosis in a variety of diseases. Cell cycle regulation ensures the correctness of DNA replication and division. These checkpoints permit progression of the cell cycle or arrest of it in response to DNA damage to provide an opportunity for DNA repair. We also observed that nicotine induces cell cycle arrest in association with increased activation of α7nAChR pathways (Figure 6). Although this study suggests that α7nAChR is probably a target for the effect of nicotine on liver cancer progression, it is in controversy with our working hypothesis and provides evidence against a direct role of nicotine in the development of liver cancer. In this study, we could not obtain more detailed mechanisms regarding other cytotoxic responses of HepG2 cells to nicotine exposure, thus further studies are needed to determine the exact intracellular signaling pathways downstream of α7nAChR in the pathogenesis of liver cancer.

## Conclusion

Altogether, it can be concluded that nicotine could lead to disturbance of the crucial equilibrium in cell death and proliferation, which may result in compensatory proliferation in the liver and eventually dysregulated increase of the cells. The findings suggest caution in the use of nicotine, as they could have a potentially detrimental effect on patients at high-risk for liver tumor development.

## Ethical Issues

The study was approved by the ethical committee of National Institute for Medical Research Development, Iran (ethical code: 1397.494).

## Conflict of Interest

The authors have no conflicts of interest to declare.

## Acknowledgments

This work was financially supported by grants from the National Institute for Medical Research Development (NIMAD), Iran (project no. 972536) and Tabriz University of Medical Sciences, Tabriz, Iran (project no. 59256). The authors want to acknowledge Dr. Ali R. Mani (University College London) for carefully reading and valuable comments on the paper.
